# The Role of *Bacopa monnieri* in Alzheimer’s Disease: Mechanisms and Potential Clinical Use—A Review

**DOI:** 10.3390/nu17223538

**Published:** 2025-11-12

**Authors:** Michał Wiciński, Anna Fajkiel-Madajczyk, Jakub Wójcicki, Mateusz Ozorowski, Monika Szambelan

**Affiliations:** Department of Pharmacology and Therapeutics, Faculty of Medicine, Collegium Medicum in Bydgoszcz, Nicolaus Copernicus University, M. Curie Skłodowskiej 9, 85-094 Bydgoszcz, Poland; michal.wicinski@cm.umk.pl (M.W.); jakub.wojcicki@cm.umk.pl (J.W.); m.ozorowski@cm.umk.pl (M.O.); monika.szambelan@cm.umk.pl (M.S.)

**Keywords:** *Bacopa monnieri*, brahmi, Alzheimer’s disease

## Abstract

Over the past few years, there has been a growing interest in traditional Ayurvedic medicine, which extensively utilizes plants. One such plant is *Bacopa monnieri*, also known as brahmi, which has gained particular popularity for its cognitive-function-enhancing properties and neuroprotective effects. Numerous studies highlight its antioxidant, anti-inflammatory, anti-stress, anti-anxiety, and even symptom-reducing properties associated with attention deficit–hyperactivity disorder (ADHD). Additionally, in vitro and in vivo studies demonstrate the potential therapeutic applications of this plant and its active compounds, particularly bacosides, in treating Alzheimer’s disease. This review aims to evaluate whether *B. monnieri* can serve as a potential therapy enhancer and slow the progression of Alzheimer’s disease. We have attempted to clarify the mechanisms of action and the possible clinical application of brahmi in this progressive neurodegenerative brain disorder.

## 1. Introduction

*B. monnieri*, also called brahmi, is a well-known herbal remedy rooted in Ayurvedic tradition [1]. This small, creeping herb features numerous branches, tiny oblong leaves, and bright purple flowers. It belongs to the Scrophulariaceae family and is regarded as a “grace” herb in Ayurveda. Naturally, brahmi thrives in wet, humid, and marshy regions across the Indian subcontinent [2]. Its rich mix of active compounds—such as alkaloids, glycosides, flavonoids, triterpenes, and saponins—has led to its widespread use in treating various health issues, especially neurological conditions like Alzheimer’s disease (AD), Parkinson’s disease, Huntington’s disease, and epilepsy [1,2].

Despite many years of research, no drug has yet been found that is completely effective and safe in preventing the progression of AD and managing its symptoms. As interest in natural medicines and plants used in traditional medicine grows, researchers are increasingly exploring their potential for treating neurodegenerative conditions. This review examines whether *B. monnieri* could act as a therapy enhancer and slow AD progression. We aim to elucidate the herb’s mechanisms of action and its potential clinical applications in the treatment of this neurodegenerative disorder. The review also highlights future research directions and key considerations for incorporating *B. monnieri* into medical practice.

## 2. AD

In 1906, the world first learned about AD. It was during the congress of German psychiatrists in Tübingen [3]. Alois Alzheimer described a case of a 50-year-old woman who, for five years until her death, was hospitalized at the Community Psychiatric Hospital in Frankfurt am Main because of the rapid progression of paranoid symptoms with associated aggression, deteriorating memory, and sleep disturbances. An autopsy of her brain revealed characteristic histological changes later known as plaques and neurofibrillary tangles. This discovery did not impress the congress attendees [4]. Over the following three years, Alzheimer and his assistant described only three additional similar cases [5]. At the start of the 20th century, life expectancy was only 45–50 years in industrialized countries [6]. With advances in medicine and increased longevity, more cases of AD have been diagnosed. The development of brain imaging techniques, such as Positron Emission Tomography (PET) and Magnetic Resonance Imaging (MRI), now enables the early detection of the disease’s initial stages [7].

### 2.1. AD Risk Factors

AD is classified into two groups: late-onset AD (LOAD) or sporadic, which affects more than 95% of AD patients, and early-onset AD (EOAD) or familial, which impacts 1–5% of patients [8]. Genetic mutations cause EOAD in three autosomal dominant genes that encode the amyloid β (Aβ) precursor protein (APP), presenilin-1 (PSEN1), and presenilin-2 (PSEN2). The PSEN1 and PSEN2 proteins are subunits of the enzyme γ-secretase, which cleaves APP [9]. Mutations in these genes lead to abnormalities, such as the overproduction of amyloid β 42 (Aβ42), which tends to aggregate [10].

The causes of sporadic AD are multifactorial. The following factors are considered: aging is the primary risk factor, along with non-genetic risks such as atherosclerosis, diabetes, lifestyle, cerebrovascular disease, and neuropsychiatric disorders [11]. Apolipoprotein E (ApoE) is involved in transporting lipids and cholesterol in the brain. Carriers of the ε4 allele of apolipoprotein have a higher risk (holders of 1 allele four times, and homozygotes up to ten times) of AD. The factors influencing the expression of this gene are not fully understood [10].

### 2.2. AD Pathogenesis and Treatment

Some hypotheses suggest that Aβ has a physiological role in regulating synaptic plasticity, such as by reducing neuronal excitatory transmission. The rising levels of Aβ oligomers and plaque formation are likely contributing factors to the neurodegeneration seen in AD [12]. Histopathological examinations of the brains of people with AD also confirm the presence of plaques consisting of Aβ, dystrophic neuritis in the cortical layer, and intracellular neurofibrillary tangles composed of hyperphosphorylated tau protein in the medial temporal lobe structures [13].

Loss of cognitive functions dependent on cholinergic conduction was one of the earliest hypotheses of AD [14]. Acetylcholinesterase (AChE) inhibitors (donepezil, galantamine, rivastigmine, and tacrine) and a low-affinity NMDA (N-methyl-D-aspartate) receptor antagonist (memantine) are medications that alleviate some symptoms but do not stop the progression of the disease [15]. Synaptic dysfunction is also associated with impaired conduction of ryanodine and calcium L-type receptors. This results in excessive release of ionized calcium intracellularly, which accelerates cell neurodegeneration and apoptosis [16].

The next step in the therapy was the synthesis of antibodies, which, after crossing the blood–brain barrier (BBB), would bind to Aβ to activate the immune response and remove Aβ from the brain. Unfortunately, most Aβ immunotherapies caused severe side effects, such as meningoencephalitis, microhemorrhage, and vasogenic edema [17]. Since the Food and Drug Administration (FDA) has approved lecanemab and donanemab (2023 and 2024, respectively), these two anti-amyloid therapies reduce Aβ and tau buildup in the brain. The result of this treatment is a slowdown in cognitive decline in AD patients with a lower risk of side effects [18]. However, using these drugs increases the risk of seizures and bleeding into the central nervous system, especially in patients taking anticoagulants [19]. There are also many studies focused on developing safe anti-tau antibodies. The aim is to eliminate tau and slow the progression of AD [20].

The autophagy–lysosomal dysfunction hypothesis posits that impaired autophagic flux and lysosomal degradation lead to the accumulation of misfolded proteins and defective organelles. The cause involves mutations or dysfunctions in endosomal–lysosomal genes (*SORL1*, *PICALM*, *BIN1*) that disrupt the transport of APP, tau, and Aβ. Animal studies have shown several possible methods of stimulating autophagocytosis. The use of rapamycin (which inhibits the mammalian target of rapamycin complex 1—mTORC1 pathway), metformin, resveratrol, and berberine (which activate AMP-activated protein kinase—AMPK), curcumin, aspirin, and gemfibrozil (which activate transcription factor EB—TFEB) induces autophagocytosis and thereby provides neuroprotective effects [21].

Pathological proteins accumulating in mitochondria lead to their dysfunction. The chain of respiratory processes and the elimination of free radicals are disturbed. The result is a decrease in energy production in the form of ATP, which counteracts the damaging effects of pathological oxygen species and mutations within mitochondrial DNA [22].

Increasing evidence from both animal and human models suggests that insulin and insulin-like growth factor (IGF) play a role in signaling cascades within the brain. They act by activating complex intracellular signaling pathways, resulting in the formation of insulin receptor substrate molecules (IRS). IRS molecules are a signal of activation of the extracellular signal-regulated kinase/mitogen-activated protein kinase (ERK/MAPK) and phosphatidylinositol-3 kinase (PI3/Akt) pathways and inhibition of glycogen synthase kinase 3β (GSK-3β) [23]. This cascade starts processes important for the proper functioning of cells and their survival. When brain cells develop insulin resistance during AD, it is referred to as type 3 diabetes. The expression of genes encoding tau protein and its subsequent phosphorylation depend on a properly functioning series of chemical reactions induced by insulin [24].

Tang et al. [25] examined the link between different medications used to treat type 2 diabetes (T2D) and the occurrence of AD and dementias. Their study found that patients with T2D treated with glucagon-like peptide-1 receptor agonists (GLP-1RAs) and sodium-glucose cotransporter 2 inhibitors (SGLT2is) had a lower risk of developing AD and related dementias compared to those on second-line glucose-lowering drugs. Therefore, the neuroprotective effects of these medications have been confirmed [25].

In response to theories that inflammation causes AD development, edaravone was developed. This is a potent antioxidant that reduces the impact of free radicals on neurons and lipid peroxidation. Edaravone is a promising drug that has the potential to slow the progression of neurodegenerative diseases by reducing glial cell overactivation and synaptic damage. However, it is still not FDA-approved [26].

**Figure 1:**
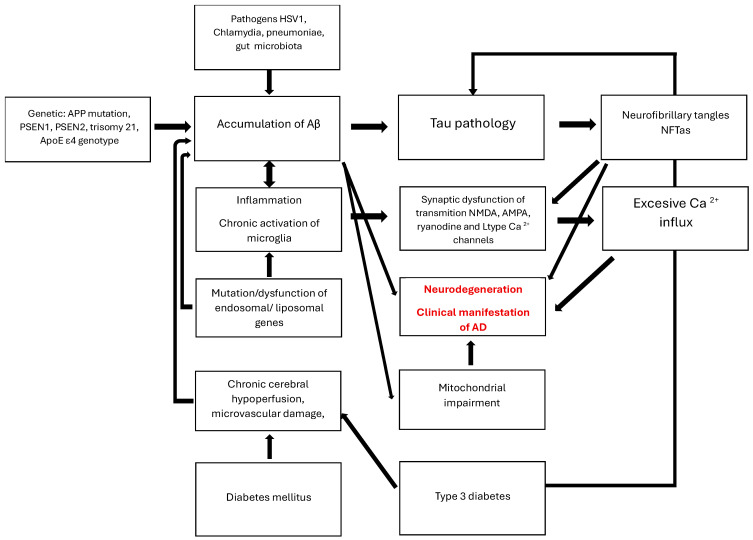
AD pathogenesis. The graphic illustrates the multifactorial pathway of neurodegenerative damage to the brain. It begins with causes such as increased production of pathological proteins, including inflammatory factors, altered gut microbiome, damaged BBB associated with diabetes and microangiopathy, as well as impaired clearance of Aβ and tau by endosomes, liposomes, and inflammation-induced glial cells. Intracellular changes impair the functions of organelles, particularly mitochondria. This secondary damage obstructs communication between neurons in the acetylcholine, glutamate, glycine, and calcium systems. An excessive release of calcium inside cells signals the initiation of apoptosis processes. Molecular damage initially manifests as a disorder of recent memory, leading to the loss of independent functioning.

Figure 1 illustrates the complexity of AD pathogenesis.

## 3. Characteristics of *B. monnieri*

### 3.1. Active Substances

Brahmi is believed to contain approximately 52 phytomolecules that may potentially interact with nearly 800 human direct receptors [27]. This substantial amount explains its widespread positive effects on the human organism. Some of the best-known active ingredients of this plant are: bacobitacin A–D, bacoside A1–A3, bacoside A4–A5, bacogenin A1–A4, bacosine, bacopaside I–II, bacopasaponin A–I, bacomosaponin A–B [28].

**Figure 2:**
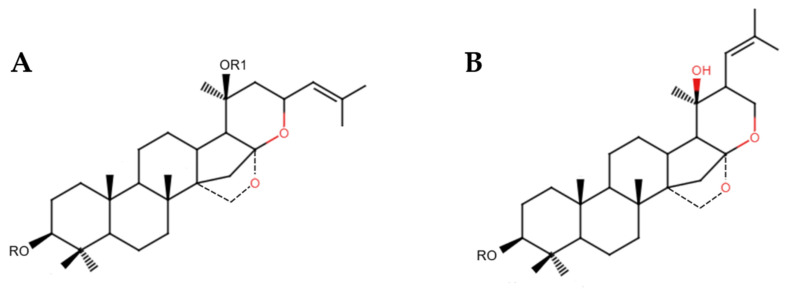
Chemical structures of (**A**) jujubogenin and (**B**) pseudojujubogenin.

Bacosides are among the most significant active compounds in *B. monnieri*, classified as dammarane-type triterpenoid saponins [29]. The main site for their biosynthesis and accumulation is the brahmi shoot region. These compounds include two isomers: jujubogenin and pseudojujubogenin (see Figure 2) [30]. They are generated through the cyclization of 2,3-oxidosqualene into a dammarane-type triterpene by dammarenediol synthase, followed by modifications via cytochrome P450 enzymes to produce the two isomers. Subsequently, uridine 5′-diphospho-glucuronosyltransferase (UGT) enzymatically converts these isomers into various bacosides [30].

Among the substances in *B. monnieri*, bacoside A stands out. It is part of the main saponin fraction, which makes up about 38% of the dry weight of the standardized methanol extract, and includes four saponin glycosides: bacopaside II, bacopaside X, bacoside A3, and bacopasaponin C [31]. Numerous studies have shown that bacosides are responsible for most of brahmi’s neuroprotective and nootropic properties [2]. Furthermore, these compounds have been demonstrated in various preclinical and clinical studies to possess anti-aging, anticancer, anticonvulsant, antidepressant, antiemetic, anti-inflammatory, and antimicrobial properties [29]. Another interesting group of substances linked to the neuroprotective effects of *B. monnieri* includes alkaloids like brahmine and herpestine. Herpestine has also been shown to influence neurotransmitters such as serotonin and dopamine, thereby aiding in the regulation of mood and cognitive function [32].

### 3.2. Molecular Mechanisms

**Figure 3:**
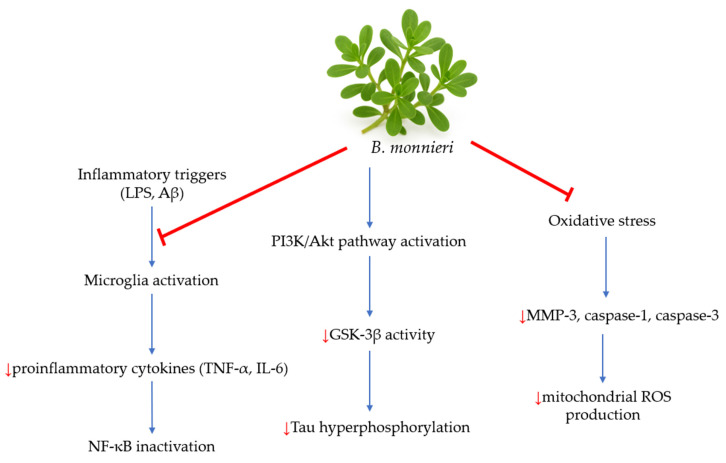
The potential mechanisms of *B. monnieri* in AD [2]. Inflammatory triggers, such as LPS and Aβ, stimulate microglia activation. *B. monnieri* modulates that activation and leads to decreased production of pro-inflammatory cytokines like TNF-α and IL-6. Inhibiting NF-κB activation reduces neuroinflammation by decreasing the expression of inflammatory genes. Additionally, this plant activates the PI3K/Akt pathway, a crucial pathway for cell survival and neuroprotection. Activation of this pathway inhibits GSK-3β, a key kinase involved in tau hyperphosphorylation in AD. *B. monnieri* also reduces oxidative stress and decreases levels of MMP-3, caspase-1, and caspase-3, which contribute to a reduction in ROS production.

Studies have demonstrated that *B. monnieri* inhibits the release of pro-inflammatory cytokines, such as tumor necrosis factor-alpha (TNF-α) and interleukin-6 (IL-6), from N9 microglial cells in vitro. Additionally, it suppresses caspase-1 and -3, as well as matrix metalloproteinase-3 (MMP-3), in a cell-free assay, indicating its potential for reducing inflammation in the central nervous system [33]. Researchers emphasize *B. monnieri*’s potent antioxidant effects, which, through the nuclear factor kappa B (NF-κB) signaling pathway and MAPKs, help combat neuroinflammation in neurodegenerative diseases [28,34]. Other studies have also shown that *B. monnieri* can inhibit tau aggregation in vitro [35]. Moreover, cells treated with brahmi exhibited lower levels of reactive oxygen species (ROS) and decreased caspase-3 activity. Immunoblot and immunofluorescence analyses revealed that *B. monnieri* acts as an antioxidant and supports the restoration of nuclear factor erythroid 2-related factor 2 (Nrf2) levels in Neuro2a cells. When Neuro2a cells were treated with *B. monnieri*, they displayed a reduction in phospho-tau load compared to those exposed to formaldehyde. Furthermore, this treatment reduced GSK-3β phosphorylation under formaldehyde stress. These results suggest the potential use of brahmi in AD [35]. Figure 3 illustrates the possible mechanisms of *B. monnieri* in AD.

Interestingly, *B. monnieri* may also have uses in treating other diseases, although not all of its mechanisms of action are currently understood. Network pharmacology indicates the potential of *B. monnieri* for treating liver cancer, which needs confirmation through in vitro and in vivo studies [36]. In a study using an ethanolic extract of *B. monnieri*, the extract was found to inhibit proliferation and induce apoptosis by reducing oxidative stress in the human liver cancer cell line (HepG2). These studies have confirmed the herb’s potential hepatoprotective properties [37].

**Figure 4:**
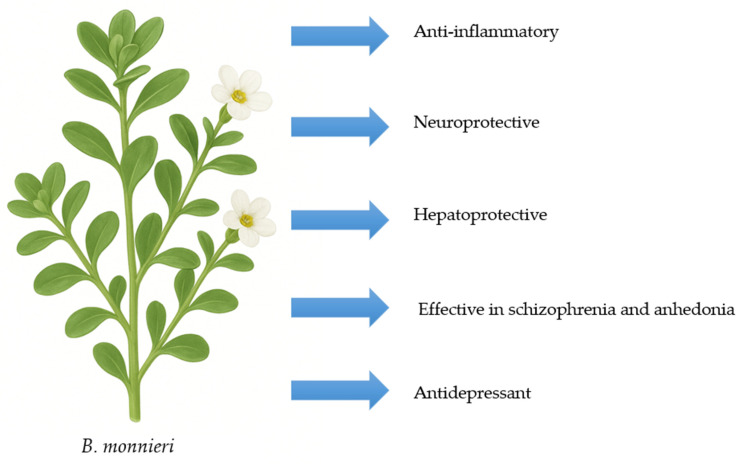
Potential medicinal properties of *B. monnieri*.

Moreover, brahmi seems effective in treating mental illnesses. A systematic review by Ayilara et al. [38] found that *B. monnieri* can reduce positive, negative, and cognitive symptoms of schizophrenia. Its mechanism involves altering the glutamatergic pathway and GABAergic transmission, lowering malondialdehyde (MDA) levels, increasing glutathione (GSH) levels, slowing AChE activity, and preserving neuronal density [38]. Research in a mouse model exploring *B. monnieri* as a potential therapy for anhedonia revealed important findings [39]. The sucrose preference test, which directly measures the ability to feel pleasure, showed that *B. monnieri* extract prevented the decrease in sucrose consumption caused by lipopolysaccharide (LPS). Additionally, brahmi significantly lowered plasma levels of cytokines, cortisol, and artemisinin that depended on LPS, while it increased levels of brain-derived neurotrophic factor (BDNF) [39]. Another study using an animal model found that *B. monnieri* improves depression similarly to citalopram in cases of reserpine-induced depression [40]. Figure 4 shows the potential medicinal properties of *B. monnieri*.

Despite numerous studies, not all of the molecular mechanisms by which *B. monnieri* acts are fully understood. Still, due to its rich concentration of active compounds, the plant remains a promising subject for research.

### 3.3. Safety and Toxicity

*B. monnieri* is a non-toxic plant. No significant adverse effects have been reported within the wide range of doses used (300–600 mg). It therefore appears that this plant has a high safety profile. Additionally, in vitro studies on rats have shown that *B. monnieri* does not cause chronic toxicity across a broad dose range (30, 60, 300, and 1500 mg/kg/day of *B. monnieri* extract) over a 270-day period. In most studies, no serious adverse effects were observed, except for gastrointestinal disorders [31]. Nevertheless, it is challenging to determine the effects of long-term use of *B. monnieri* extract.

Research indicates that *B. monnieri* extracts might affect herb–drug interactions by inhibiting various cytochrome isoenzymes, including CYP2C9, CYP2C19, CYP1A2, CYP2D6, and CYP3A4. Using *B. monnieri* alongside antidepressants, particularly agomelatine and moclobemide, appears especially risky. *B. monnieri* combined with agomelatine led to back pain and excessive sweating, while there have been cases of myocardial infarction linked to moclobemide [41]. There was also a report of a 58-year-old patient with Sjogren’s syndrome, taking *B. monnieri* and cevimeline simultaneously, who experienced symptoms of cholinergic toxicity, such as excessive sweating, malaise, nausea, and rapid heartbeat. This seems related to the inhibition of CYP3A4 and CYP2D6, which are involved in metabolizing the drugs. After discontinuing the supplement, the patient’s condition improved [42].

## 4. *B. monnieri* in AD

Some in silico research suggests that brahmi’s active compounds may have an affinity for hyperphosphorylated tau protein and could prevent its tangle formation [43]. However, both the composition and the entire bioprocess of cultivating the plants and extracting active compounds are essential to the extract’s effectiveness as an anti-AD agent [44]. Regarding the pathophysiology of AD, the primary actions of bacosides and other active compounds from *B. monnieri* include anti-inflammatory and antioxidant effects, reducing mitochondrial dysfunction, and decreasing Aβ aggregation and toxicity [45,46,47].

### 4.1. Preclinical Trials

Nemetchek et al. [33] investigated different types of extracts from *B. monnieri*, including tea, infusion, alkaloid, and bacoside A, derived from an ethanol extract of brahmi. Their experiments on the N9 microglial cell line from CBA mice demonstrated that both the infusion and alkaloid forms significantly (20%) reduced the secretion of TNF-α and IL-6 compared to LPS-activated microglia. Bacoside A did not decrease the secretion of these pro-inflammatory molecules. An interesting aspect of their research is that all types of extracts similarly inhibited (almost as effectively as a specific inhibitor) the key factors involved in neuroinflammation and neurodegeneration, namely caspase-1, caspase-3, and MMP-3 [33].

Brimson et al. [48], in their in vitro study on the HT-22 cell line and wild *Caenorhabditis elegans*, also observed the positive influence of brahmi extract. It was primarily expressed through an increase in mitochondrial membrane integrity and a reduction in mitochondrial stress within HT-22 cells exposed to a 5 mM glutamate solution. The extracts being compared were hexane, dichloromethane, and ethanol extracts, with the ethanol extract showing the poorest influence among them. However, for those authors, the reduction in ROS remained unclear [48]. Another interesting observation made by Brimson et al. [48] is that, after glutamate treatment, there was a 50% increase in ERP57 and CHOP protein expression, which was restored to control levels by co-treatment with *B. monnieri* extract. In the studied population of *C. elegans*, scientists found that a brahmi hexane extract concentration of 7–10 µg/mL had a positive effect on survival compared to the control group. At the same time, there was no significant increase in the 3 µg/mL group, and a decrease at concentrations higher than 10 µg/mL. However, the reduction caused by higher concentrations was not statistically significant. It was also confirmed by intestinal autofluorescence assessment—an indicator of aging and oxidative stress in *C. elegans* and many other organisms—that these aspects were reduced in the group treated with 10 µg/mL extract [48]. A study with *Drosophila melanogaster* showed that *B. monnieri* supply decreases the activity of succinate dehydrogenase and protects cells from oxidative stress caused by paraquat exposure [49].

Additionally, Witter et al. [47] compared various medicinal plants and nutraceuticals, confirming that *B. monnieri* extract inhibits lyophilized Methionine Aβ 1–40 (MAβ40) fibrillation in their in vitro tests. Furthermore, besides the antioxidative effect, Palollathil et al. [50] have stated that brahmi extract may restore the function of many essential proteins involved in sterol and cholesterol biosynthesis, oxygen binding, and oxidoreductase activity that Aβ mainly suppresses.

Interestingly, Petcharat et al. [51] demonstrated that *B. monnieri* extract at concentrations of 100 µg/mL and 250 µg/mL did not cause neurotoxicity in SH-SY5Y neuroblastoma cells, while also increasing the survival rate in cells exposed to 100 µg/mL tert-butyl hydroperoxide (a pro-oxidant). However, the researchers’ further investigation concluded that the protective effects of brahmi extract are related to the activation of the ERK1/2 pathway and the PI3K pathway, both of which are responsible for reducing apoptosis [51].

Furthermore, Roy et al. [52] demonstrated in their in silico study that certain bioactive brahmi phytochemicals, such as bacopasaponin G and bacopasaponin N2, exhibit more favorable binding energy values with caspase-3 and tau protein kinase (TPK) I receptors than the standard anti-AD drug donepezil.

The findings suggest that *B. monnieri* possesses properties that mitigate oxidative stress, inflammation, and mitochondrial dysfunction. It also inhibits Aβ aggregation, a key factor in neurodegenerative diseases such as AD. These qualities appear particularly vital for slowing AD progression, though they need to be confirmed through clinical trials. Table 1 shows the results of preclinical trials described above.

### 4.2. Clinical Trials

Abdul Manap et al. [45] highlighted brahmi’s positive effects on time, place, and personal orientation, as well as attention and language skills, in geriatric patients with AD, along with test results for students. They also noted mechanisms such as normalization of ATPase activity and inhibition of lipid peroxidation in the hippocampus, prefrontal cortex, and striatum regions of the cerebrum in rats [45]. In another study, it was observed that *B. monnieri* extract may reduce both an acute type of oxidative stress and GSH and thiol levels, while increasing catalase activity [49]. Some studies have reported its positive influence on mice exposed to herbicides (paraquat), reducing oxidative stress and reversing motor and gait impairments, as well as decreasing behavioral abnormalities and normalizing dopamine levels and cholinergic activity [49].

A prospective, cohort, non-comparative, multicenter trial conducted by Seth et al. [53] included 98 males and females aged 50 years or older experiencing forgetfulness, disorientation, difficulty concentrating, and other cognitive issues. The trial confirmed a statistically significant positive effect of a commercial dietary supplement (Illumina^®^, San Diego, CA, USA) containing 100 mg of 20% dry brahmi extract (20 mg bacosides) on 11 measured aspects, especially in word recall and recognition, which are common challenges in AD [53]. However, Basheer et al. [54], in a systematic review of randomized controlled trials, found no difference between *B. monnieri* and either placebo or donepezil in treating AD or mild cognitive impairment. No significant safety concerns were reported in the studies included in this review. Additionally, the studies examined showed considerable variation in *B. monnieri* doses (from 125 mg to 500 mg twice daily), as well as differences in treatment length, follow-up periods, and outcomes. The researchers emphasized the need for clinical trials with larger participant groups [54].

Prabhakar et al. [55] compared the effectiveness of 300 mg brahmi and 10 mg donepezil treatments in 48 patients with AD in a 52-week, randomized, double-blind, parallel-group, phase 2 single-center clinical trial. After one year of treatment and observation, no significant difference was observed in the Alzheimer’s Disease Assessment Scale-Cognitive subscale and the Postgraduate Institute Memory Scale, which is an encouraging outcome [55].

Between June 2015 and May 2016, Mishra et al. [56] conducted a study on twelve patients of either sex, aged 18 years or older, who were suffering from dementia. The patients received 250 mg of brahmi extract twice daily for three months. Their small study showed a highly statistically significant reduction in scores on the Global Deterioration Scale, suggesting that *B. monnieri* extract may be very effective in treating dementias [56]. Additionally, Raghav et al. [57] aimed to explore alternative treatments for neurological deficits. In their double-blind, placebo-controlled, randomized study, they investigated the performance of a standardized *B. monnieri* extract in a group of 35 male and female subjects aged 55 to 70. The participants had age-related memory impairment without evidence of any type of dementia. Analyzing six aspects—mental control, logical memory, digit forward, digit backward, visual reproduction, and paired associate learning—with an overall memory score, the researchers concluded that standardized brahmi extract improved three (mental control, logical memory, and paired associate learning) out of six aspects [57]. In the digit forward test, a slight improvement was observed in the brahmi-treated group; however, it was not statistically significant.

Another important aspect of the clinical use of brahmi extract in AD patients is its safety profile. Compared to memantine, which is considered a safe drug, some authors note the presence of adverse effects in approximately 2% of memantine-treated patients [58]. Meanwhile, clinical trials with *B. monnieri* have reported similar or even lower frequencies of adverse effects [54,55,56].

Although in vitro results seemed promising, the clinical studies mentioned above cast doubt on using brahmi to treat AD. The studies make it hard to determine an effective therapeutic dose and treatment duration. Nevertheless, it is noteworthy that some subjects experienced improvements in cognitive function after using *B. monnieri*. Table 2 summarizes the molecular mechanisms of *B. monnieri* in AD.

## 5. Limitations and Directions for Future Research

The use of *B. monnieri* in medicine is limited for several reasons. There are no clear, large-scale human clinical trials. Most studies were conducted in vitro, in small human populations, or on animals. Different doses of *B. monnieri* were administered at various intervals, and the population sizes varied. Another factor limiting the potential use of brahmi in clinical practice is its low bioavailability. Due to the poor permeability of the bacosides and alkaloids of *B. monnieri* across the BBB, as well as the drug’s poor bioavailability to the central nervous system, the use of brahmi as a therapeutic molecule in clinical practice is severely limited [59].

One method that could be used in the future to increase the bioavailability of *B. monnieri* active substances is the creation of an appropriate polymer and encapsulation system with maximum bioavailability and a long retention time. The encapsulation process with polymeric nanoparticles has more advantages over other nanoparticle systems. Polymeric nanoparticles offer controlled drug release, efficient targeted drug delivery, ease of biodegradability, and excellent biocompatibility with tissues and cells. They also improve the plasma half-life, stability, and solubility and reduce the immunogenicity of drugs [60,61]. The most commonly used encapsulation polymers include poly(L-lactic acid) (PLA), poly(D, L-lactide-co-glycolide) (PLGA), polyethylene glycol (PEG), poly(ε-caprolactone) (PCL), polyalkylcyanoacrylates, chitosan, gelatin, and hyaluronic acid [62]. Poly(lactic-glycolic acid) (PLGA) nanoparticles are one of the most promising drug delivery systems for crossing the BBB. PLGA has excellent biocompatibility, and when exposed to the human physical environment, it is hydrolyzed to lactic acid and glycolic acid, which are naturally occurring metabolites. PEGylation contributes to increased water solubility and stability, prevents intermolecular aggregation, reduces immunogenicity, and prolongs the systemic circulation time of the compound. PEG is often combined with PLGA to obtain similar beneficial effects [63]. There are only very limited studies on the nanoencapsulation of bacoside A or bacopaside I, especially in the context of neuroprotection.

Furthermore, preparations containing brahmi vary in quality and concentration of active ingredients. The lack of standardization and clear dosing guidelines restricts the medical use of these compounds. Another concern is the limited number of studies on how brahmi’s active compounds interact with medications.

Many health authorities, including the FDA and the European Medicines Agency (EMA), have not approved *B. monnieri* as a drug; instead, they recognize it only as a dietary supplement. A key issue with herbal medicinal products is accurately identifying plant material, which involves avoiding herbs contaminated with toxins, pesticides, and heavy metals, as well as considering their potential interactions with standard pharmacotherapy [2]. It is possible that in the future, *B. monnieri* will be incorporated into the diet in standardized doses. However, at present, its use in treating AD requires further research, safety evidence, and demonstration of efficacy.

## 6. Conclusions

Based on the preclinical and clinical studies, it can be concluded that *B. monnieri* shows promising potential as a supportive agent in the treatment of AD. Its neuroprotective effect is primarily related to its antioxidant and anti-inflammatory properties, as well as its ability to modulate the cholinergic system and inhibit Aβ aggregation. The results of the studies suggest that supplementation with brahmi extract may improve cognitive functions, particularly in the areas of working memory and learning, which are of particular importance in the context of the progressive cognitive decline associated with AD. Despite the positive results of some studies, further well-designed clinical studies on larger patient populations are necessary to unequivocally confirm the efficacy and safety of *B. monnieri* in the treatment of AD. It is also crucial to establish optimal doses and standardize the extraction of *B. monnieri* extracts, as well as possible interactions with other drugs used in the treatment of AD.

## Figures and Tables

**Table 1:** The results of preclinical trials with *B. monnieri*.

Author	Study Type	Study Group	Results
Nemetchek et al. [33]	In vitro	N9 microglial cell line from CBA mice	Different types of *B. monnieri* extracts effectively inhibit the release of TNF-α and IL-6 from cells. Inhibition of caspase-1 and -3, and MMP-3.
Brimson et al. [48]	In vitro	HT-22 cell line and wild *C. elegans*	Anti-glutamate toxicity action.
Witter et al. [47]	In vitro	Lyophilized Aβ40 and lyophilized MAβ40	Inhibition of Aβ fibrillation.
Palollathil et al. [50]	In vitro	IMR-32 cells (ATCC CCL-127)	Antioxidative action via free radical scavenging, neuroprotective effect, improvement in extracellular matrix organization, IL-4, and IL-13 signaling.
Petcharat et al. [51]	In vitro	SH-SY5Y neuroblastoma cells	Increase in ERK1/2 and Akt phosphorylation.
Roy et al. [52]	In silico	N/A	Bacosaponines demonstrate higher receptor affinity compared to donepezil.

N/A—not available.

**Table 2:** *B. monnieri* in AD—molecular mechanisms.

Factor	Effect of *B. monnieri*	Mechanism	References
Oxydative stress	↓	Lipid peroxidation decreases, and ROS scavenging.	[45]
Tau protein tangling	↓	Inhibition occurs by interacting with the R2 repeat domain of the hyperphosphorylated protein.	[43]
Aβ aggregation	↓	The binding of bacoside A to amyloid oligomers prevents it from aggregating.	[45,46,47]
Neuroinflammation	↓	Decrease in TNF-α and IL-6 levels. Inhibition of caspase-1, caspase-3 and MMP-3.	[33,45,46,47]
Neurodegeneration	↓	Decrease in apoptosis by increasing activation (phosphorylation) of ERK1/2 and PI3K pathways.	[33,51]

↓ Decrease.

## Data Availability

No new data were created or analyzed in this study.
